# Circulating total bilirubin and risk of non-alcoholic fatty liver disease in the PREVEND study: observational findings and a Mendelian randomization study

**DOI:** 10.1007/s10654-019-00589-0

**Published:** 2019-11-26

**Authors:** Setor K. Kunutsor, Monika Frysz, Niek Verweij, Lyanne M. Kieneker, Stephan J. L. Bakker, Robin P. F. Dullaart

**Affiliations:** 1 National Institute for Health Research Bristol Biomedical Research Centre, University Hospitals Bristol NHS Foundation Trust and University of Bristol, Bristol, UK; 2 Musculoskeletal Research Unit, Translational Health Sciences, Bristol Medical School, University of Bristol, Learning and Research Building (Level 1), Southmead Hospital, Bristol, BS10 5NB UK; 3 Department of Cardiology, University of Groningen and University Medical Center, Groningen, The Netherlands; 4 Department of Nephrology Medicine, University of Groningen and University Medical Center, Groningen, The Netherlands; 5 Department of Endocrinology, University of Groningen and University Medical Center, Groningen, The Netherlands

**Keywords:** Total bilirubin, Non-alcoholic fatty liver disease, Cohort study, Mendelian randomization

## Abstract

The relationship between circulating total bilirubin and incident non-alcoholic fatty liver disease (NAFLD) is uncertain. We aimed to assess the association of total bilirubin with the risk of new-onset NAFLD and investigate any causal relevance to the association using a Mendelian randomization (MR) study. Plasma total bilirubin levels were measured at baseline in the PREVEND prospective study of 3824 participants (aged 28–75 years) without pre-existing cardiovascular disease or NAFLD. Incident NAFLD was estimated using the biomarker-based algorithms, fatty liver index (FLI) and hepatic steatosis index (HSI). Odds ratios (ORs) (95% confidence intervals) for NAFLD were assessed. The genetic variant rs6742078 located in the UDP-glucuronosyltransferase (UGT1A1) locus was used as an instrumental variable. Participants were followed up for a mean duration of 4.2 years. The multivariable adjusted OR (95% CIs) for NAFLD as estimated by FLI (434 cases) was 0.82 (0.73–0.92; *p *= 0.001) per 1 standard deviation (SD) change in log_e_ total bilirubin. The corresponding adjusted OR (95% CIs) for NAFLD as estimated by HSI (452 cases) was 0.87 (0.78–0.97; *p *= 0.012). The rs6742078 variant explained 20% of bilirubin variation. The ORs (95% CIs) for a 1 SD genetically elevated total bilirubin level was 0.98 (0.69–1.38; *p *= 0.900) for FLI and 1.14 (0.81–1.59; *p *= 0.451) for HSI. Elevated levels of total bilirubin were not causally associated with decreased risk of NAFLD based on MR analysis. The observational association may be driven by biases such as unmeasured confounding and/or reverse causation. However, due to low statistical power, larger-scale investigations are necessary to draw definitive conclusions.

## Introduction

Circulating total bilirubin has been consistently shown to be inversely and independently associated with adverse cardiometabolic outcomes such as cardiovascular disease (CVD), hypertension and type 2 diabetes [1–3]. Though a causal association has been demonstrated for total bilirubin and type 2 diabetes [4], there is no strong evidence for a causal association between total bilirubin levels and CVD [5, 6]. Nonalcoholic fatty liver disease (NAFLD), emerging as the most common cause of chronic liver disease in the developed world [7], is a cardiometabolic condition which is characterized by hepatic steatosis with varying degrees of necroinflammation and fibrosis [7]. In the absence of the reference standard—liver biopsy [8], the diagnosis of NAFLD is commonly based on (1) imaging techniques [i.e., ultrasonography, computed tomography (CT) scan, or magnetic resonance imaging (MRI)] confirming the presence of fat infiltration of the liver, (2) exclusion of other liver diseases of other aetiology and (3) in the absence of substantial alcohol intake [9]. Due to the high costs associated with these imaging techniques and their unsuitability for use in large-scale population-based studies, a number of biomarker-based algorithms have been developed to aid the diagnosis of NAFLD. The fatty liver index (FLI) [10] and the hepatic steatosis index (HSI) [11] are based on easily accessible variables and have been reported to have good diagnostic accuracies for NAFLD [10–12].

Given the existence of a strong link between total bilirubin and adverse cardiometabolic outcomes, which has been attributed to the antioxidant [13, 14], anti-inflammatory [15] and antiatherogenic properties of bilirubin; [16] there have been suggestions that circulating total bilirubin might also be associated with a reduced risk of NAFLD. Indeed, a number of observational studies have reported on the associations between bilirubin and NAFLD over recent years. However, these were based on cross-sectional or case–control study designs limited by lack of temporality, paediatric populations, selected patients with pre-existing disease or were unable to demonstrate associations specifically between total circulating bilirubin and NAFLD risk [17–22]. Therefore uncertainty remains regarding the nature and magnitude of the prospective association between total bilirubin and NAFLD. With the ongoing debate on the potential value of using circulating total bilirubin to prevent and treat cardiometabolic outcomes [23–25], it will be clinically useful if circulating total bilirubin is shown to contribute to the development of NAFLD. In this context, we aimed to quantify the nature and magnitude of the prospective association between total bilirubin and the risk of NAFLD (as estimated by these two indices—FLI and HSI) in a general population sample who were free from pre-existing disease (NAFLD, CVD, and malignancy) at baseline. Since observational epidemiological studies are beset by several biases such as residual confounding, reverse causation, and regression dilution [26–29], we utilized a Mendelian randomisation (MR) to assess if there is a causal relevance to the association using the well-known rs6742078 variant in the *UGT1A1* gene [6, 30].

## Materials and methods

### Study population

We conducted this study according to STROBE (STrengthening the Reporting of OBservational studies in Epidemiology) guidelines for reporting observational studies in epidemiology (“Appendix 1”) [31]. Participants for the current analysis were part of the ongoing Prevention of Renal and Vascular End-stage Disease (PREVEND) study, a large-scale general population-based observational cohort study which began in 1997 in the Netherlands. The PREVEND study was designed to investigate the natural course of urinary albumin excretion and its relationship to renal and CVD progression. The study design and recruitment procedures have been described in detail in several previous reports [2, 32]. Briefly, 8592 inhabitants aged 28–75 years living in the city of Groningen, the Netherlands were recruited into the PREVEND study with baseline measurements performed between 1997 and 1998. For the present analysis, we excluded participants (1) with a prevalent history of CVD, renal disease, malignancy, or NAFLD (for the analysis of FLI outcome, participants with prevalent NAFLD as measured by FLI were excluded and vice versa for HSI) and (2) with excessive alcohol use (defined as four or more drinks per day), which left a cohort of 3824 participants (based on FLI) with non-missing information on total bilirubin levels, relevant covariates, and outcomes. Of these participants, 1610 individuals had complete phenotypic and genotypic data. The PREVEND study complies with the Declaration of Helsinki and was approved by the medical ethics committee of the University Medical Center Groningen. All participants provided written informed consent for voluntary participation.

### Risk factor assessment

For the assessment of baseline data on sociographic characteristics, physical measurements, medical history, and use of medication, participants completed two outpatient visits. Further information on medication use was complemented using data from all community pharmacies in the city of Groningen and this covers complete information on drug use in 95% of PREVEND participants. Venous blood was obtained from participants after an overnight fast and 15 min of rest. Plasma samples were prepared by centrifugation at 4 °C. Blood lipids (total cholesterol, high-density lipoprotein cholesterol (HDL-C), and triglycerides (TGs)), high sensitivity C-reactive protein (hsCRP), serum creatinine, and serum cystatin C were measured using standard laboratory protocols previously described [33–35]. Total bilirubin was measured using a colorimetric assay (2,4-dicholoraniline reaction; Merck MEGA, Darmstadt, Germany), with the detection limit being 1.0 µmol/L. The inter-assay coefficients of variation were 3.8% and 2.9% in the lower normal and higher normal range respectively. The mean of two 24-h urine collections was used to estimate urinary albumin excretion (UAE) and its concentration determined by nephelometry (BNII; Dade Behring Diagnostic, Marburg, Germany). Serum liver aminotransferase (alanine aminotransferase, ALT and aspartate aminotransferase, AST) activities were measured using the standardized kinetic method with pyridoxal phosphate activation (Roche Modular P; Roche Diagnostics, Mannheim, Germany). Serum gamma-glutamyltransferase (GGT) activity was measured by an enzymatic colorimetric method (Roche Modular P; Roche Diagnostics, Mannheim, Germany). Estimated glomerular filtration rate (eGFR), was calculated using the Chronic Kidney Disease Epidemiology Collaboration (CKD-EPI) combined creatinine-cystatin C equation [36]. Body mass index (BMI) was estimated by dividing weight measured in kilograms by the square of height in meters.

### NAFLD ascertainment

The FLI was calculated based on the report by Bedogni et al. [10] using the following formula:

$${\text{FLI}} = [{\text{e}}^{{(0.953 \times \ln \left( {\text{TG}} \right) + 0.139 \times {\text{BMI}} + 0.718 \times \ln \left( {\text{GGT}} \right) + 0.053 \times {\text{WC}} - 15.745)}} ] / [1 + {\text{e}}^{{(0.953 \times \ln \left( {\text{TG}} \right) + 0.139 \times {\text{BMI}} + 0.718 \times \ln \left( {\text{GGT}} \right) + 0.053 \times {\text{WC}} - 15.745)}} ]\times 100$$

The FLI ranges from 0 to 100, with FLI < 30 ruling out (sensitivity = 87%) and FLI ≥ 60 ruling in fatty liver disease (specificity = 86%) with a good diagnostic accuracy of 0.84 (95% CI 0.81–0.87).

The HSI was estimated using the following formula as reported by Lee et al.: [11]

$$\begin{aligned} {\text{HSI}} & = 8 \times {\text{ALT}}/{\text{AST ratio}} + {\text{BMI }}\left( { + 2,{\text{if diabetes}}; + 2,{\text{if female}}} \right); \\ & \quad {\text{with HSI values}} < 30{\text{ and}} > 36{\text{ ruling out and ruling in fatty liver respectively}}. \\ \end{aligned}$$

### Genotyping

Genotyping was performed using Illumina HumanCytoSNP-12 arrays. SNPs were called using Illumina Genome Studio software. SNPs were excluded with minor allele frequency < 0.01, call rate < 0.95, or deviation from Hardy–Weinberg equilibrium (*p *< 1×10 − 5). The rs6742078 SNP was a suitable instrumental variable for the present analyses, given its robust specificity for circulating total bilirubin levels (explaining up to 45% of the variation in circulating bilirubin levels [37]) and its use in previous studies to assess the causal relevance of total bilirubin to several disease outcomes [5, 6, 30].

### Statistical analyses

#### Observational analyses

Skewed variables (total bilirubin, ALT, AST, TGs, hsCRP, and UAE) were natural log-transformed to approximate normal distributions. Descriptive analyses were used to summarize baseline characteristics of participants overall and according to NAFLD outcomes. Partial correlation coefficients adjusted for age and sex were calculated to assess cross-sectional associations of total bilirubin levels with risk markers for NAFLD. Logistic regression was used to examine the association of total bilirubin with new-onset NAFLD as measured by FLI or HSI (observed effect of bilirubin on NAFLD). The odds ratio (OR) with 95% confidence intervals (CIs) for the risk of NAFLD was calculated per 1 standard deviation, SD higher log_e_ total bilirubin levels. In subsidiary analyses, total bilirubin was modeled as tertiles defined according to its baseline distribution. The SD of baseline log_e_ total bilirubin level was 0.42 (equivalent to 1.5-fold higher circulating total bilirubin level, as e^0.42^ = 1.52). Odds ratios were progressively adjusted for in four models: (Model 1) age and sex; (Model 2) plus smoking status, systolic blood pressure (SBP), total cholesterol, and HDL-C; (Model 3) plus alcohol consumption, glucose, eGFR, and UAE; and (Model 4) plus hsCRP. Confounders were selected based on their known associations with NAFLD and observed associations with total bilirubin using the available data [38] and evidence from previous research [1, 2]. We used interaction tests to assess statistical evidence of effect modification by sex on the association. To minimise bias due to potential reverse causation, we carried out sensitivity analyses that excluded participants with a history of diabetes at baseline, participants on regular statin medication or participants with potential Gilbert’s syndrome (defined as defined as total bilirubin > 34.2 µmol/L, AST < 80 IU/L, ALT < 80 IU/L, and GGT < 80 IU/L) [1].

#### Mendelian randomization

To assess for pleiotropy, we tested whether rs6742078 was associated with relevant risk markers which might confound the relationship between total bilirubin and NAFLD. We assessed the association between rs6742078 and log_e_ total bilirubin using linear regression under the assumption of an additive genetic model, with adjustment for covariates used in the observational analysis between total bilirubin and NAFLD. This analysis provided the number of SDs above log_e_ total bilirubin per each copy increase in number of the T allele. In order to estimate the causal effect of bilirubin on NAFLD, we used the two-stage least squares (2SLS) method. The first stage of the 2SLS method is to examine the association between rs6742078 and total bilirubin by means of linear regression (model 1) and then saving the predicted values and the residuals. In the second stage, the predicted values of total bilirubin from model 1 are used as covariates, with NAFLD as a dependent variable in a logistic regression, which provides the MR estimate. Estimates of power for the MR analysis on NAFLD employed an online power calculator ([http:/cnsgenomics.com/shiny/mRnd/]). We used the genetic sample size and case/control ratios together with the proportion of variance of total bilirubin explained by rs6742078. Using PREVEND data, we had 20% power to detect a causal association of bilirubin on NAFLD, similar in magnitude to the fully adjusted observational OR of 0.82. All statistical analyses were conducted using Stata version 15 (Stata Corp, College Station, Texas, USA).

## Results

### Baseline characteristics and correlates of total bilirubin

Baseline descriptive characteristics of study participants as well as cross-sectional correlates of total bilirubin are shown in Table 1. The overall mean age of participants at study entry was 47 (SD 12) years and 40.6% were women. The mean (SD) of log_e_ total bilirubin level was 1.96 (0.42) µmol/l. There were weak and inverse correlations of log_e_ total bilirubin levels with physical measures (BMI and waist circumference), lipids (total cholesterol and TGs), and glucose. There were weak positive correlations with HDL-C and the liver aminotransferases. There was an inverse correlation with log_e_ hsCRP (r = − 0.24). Baseline total bilirubin levels were higher by 28% in men compared with women. The levels were lower by 12% in the combined group of current and former smokers compared with non-current smokers (Table 2). “Appendices 2–3” show baseline characteristics according to the development of NAFLD. Except for history of diabetes and smoking status, there were significant differences in baseline clinically relevant subgroups and levels of risk markers between participants who did and did not develop NAFLD (for both indices) during follow-up.

**Table 1:** Baseline characteristics and cross-sectional correlates of total bilirubin

	Overall (*N* = 3824) Mean (SD) or median (IQR) or n (%)	Partial correlation r (95% CI)^a^	Percentage difference (95% CI) in total bilirubin levels per 1 SD higher or compared to reference category of correlate^b^
Total bilirubin (µmol/l)	7 (5–9)	–	–
Sex			
Female	2273 (59.4)	–	Ref
Male	1551 (40.6)	–	28% (25, 31)***
*Questionnaire*			
Age at survey (years)	47 (12)	− 0.02 (− 0.06, 0.01)	− 1% (− 2, 0)
History of diabetes			
No	3801 (99.4)	–	Ref
Yes	23 (0.6)	–	− 8% (− 22, 8)
Smoking status			
Non-smokers	1309 (34.2)	–	Ref
Current and former smokers	2515 (65.8)	–	− 12% (− 14, − 9)***
Alcohol consumption			
Non-consumers	886 (23.2)	–	Ref
Current consumers	2938 (76.8)	–	3% (0, 7)*
*Physical measurements*			
BMI (kg/m^2^)	24.4 (2.8)	− 0.11 (− 0.15, − 0.09)***	− 5% (− 6, − 3)***
Waist circumference (cm)	82.6 (9.5)	− 0.08 (− 0.11, − 0.05)***	− 4% (− 5, − 2)***
SBP (mmHg)	123 (18)	− 0.01 (− 0.04, 0.02)	− 1% (− 2, 1)
DBP (mmHg)	71 (9)	− 0.03 (− 0.06, 0.00)	− 1% (− 3, 0)
*Lipid, metabolic, inflammatory, liver, and renal markers*			
Total cholesterol (mmol/l)	5.44 (1.07)	− 0.09 (− 0.13, − 0.06)*	− 4% (− 5, − 3)*
HDL-C (mmol/l)	1.43 (0.39)	0.10 (0.07, 0.13)***	5% (3, 6)***
Triglycerides (mmol/l)	0.98 (0.75–1.31)	− 0.15 (− 0.18, − 0.12)***	6% (− 7, − 5)***
Glucose (mmol/l)	4.60 (0.73)	− 0.05 (− 0.08, − 0.02)*	− 2% (− 3, − 1)*
GGT (U/L)	19 (14–27)	− 0.02 (− 0.05, 0.01)	− 1% (− 2, 0)
ALT (U/L)	18 (14–23)	0.03 (0.00, 0.06)*	1% (0, 3)*
AST (U/L)	23 (20–27)	0.09 (0.06, 0.13)***	4% (3, 6)***
hsCRP (mg/l)	0.90 (0.41–2.12)	− 0.24 (− 0.27, − 0.21)***	− 9% (− 10, − 8)***
eGFR (ml/min/1.73 m^2^)	90.8 (15.0)	− 0.01 (− 0.04, 0.02)	− 0% (− 2, 1)
UAE (mg/24 h)	8.14 (5.91–12.53)	− 0.01 (− 0.04, 0.03)	1% (− 1, 2)

*ALT* alanine aminotransferase, *AST* aspartate aminotransferase, *BMI* body mass index, *DBP* diastolic blood pressure, *eGFR* estimated glomerular filtration rate (as calculated using the Chronic Kidney Disease Epidemiology Collaboration combined creatinine-cystatin C equation), *GGT* gamma-glutamyltransferase, *HDL-C* high-density lipoprotein cholesterol, *hsCRP* high-sensitivity C-reactive protein, *Ref* reference, *SD* standard deviation, *SBP* systolic blood pressure, *UAE* urinary albumin excretion

^a^Partial correlation coefficients between log_e_ total bilirubin and each of the row variables adjusted for age and sex

^b^Percentage change in total bilirubin levels per 1 SD increase in the row variable (or for categorical variables, the percentage difference in mean total bilirubin levels for the category versus the reference) adjusted for age and sex

Asterisks indicate the level of statistical significance: * *p* < 0.05; ** *p* < 0.01; *** *p* < 0.001

**Table 2:** Association of baseline total bilirubin with incident NAFLD as measured by FLI

Total bilirubin level (µmol/l)	Events/total	Model 1		Model 2		Model 3		Model 4	
		OR (95% CI)	*p* value	OR (95% CI)	*p* value	OR (95% CI)	*p* value	OR (95% CI)	*p* value
Per 1 SD increase	434/3824	0.72 (0.64 to 0.80)	< 0.001	0.78 (0.69 to 0.87)	< 0.001	0.77 (0.69 to 0.87)	< 0.001	0.82 (0.73 to 0.92)	0.001
T1 (0.95–6)	208/1627	Ref.		Ref.		Ref.		Ref.	
T2 (7, 8)	116/1024	0.70 (0.55 to 0.90)	0.006	0.85 (0.65 to 1.10)	0.209	0.85 (0.66 to 1.11)	0.232	0.92 (0.71 to 1.20)	0.536
T3 (≥ 9)	110/1173	0.51 (0.39 to 0.66)	< 0.001	0.62 (0.47 to 0.81)	< 0.001	0.61 (0.47 to 0.80)	< 0.001	0.69 (0.52 to 0.91)	0.008

2420 participants with prevalent NAFLD as measured by FLI were excluded

*CI* confidence interval, *FLI* fatty liver index, *NAFLD* non-alcoholic fatty liver disease, *OR* odds ratio, *SD* standard deviation, *T* tertile

Model 1: Age and sex

Model 2: Model 1 plus smoking status, systolic blood pressure, total cholesterol, and high-density lipoprotein cholesterol

Model 3: Model 2 plus alcohol consumption, glucose, estimated glomerular filtration rate, and log_e_ urinary albumin excretion

Model 4: Model 3 plus log_e_ high-sensitivity C-reactive protein

### Total bilirubin levels and risk of incident NAFLD

During a mean (SD) follow-up of 4.2 (0.4) years, 434 and 452 cases of NAFLD as estimated by FLI and HSI respectively, were recorded. The associations between total bilirubin and NAFLD as estimated by the FLI are reported in Table 2. In age and sex adjusted analysis, the OR for NAFLD (as estimated by FLI) per 1 SD change in log_e_ total bilirubin was 0.72 (95% CI 0.64–0.80; *p *< 0.001), which was minimally attenuated to 0.77 (95% CI 0.69–0.87; *p *< 0.001) after further adjustment for several established and emerging risk factors. Following additional adjustment for hsCRP, the OR was 0.82 (95% CI 0.73–0.92; *p *< 0.001). In analyses that compared the top versus bottom tertiles of total bilirubin, the inverse associations between total bilirubin and NAFLD were evident (Table 2).

Odds ratios for the associations between total bilirubin and NAFLD as estimated by the HSI are reported in Table 3. The age- and sex-adjusted OR for NAFLD per 1 SD change in log_e_ total bilirubin was 0.79 (95% CI 0.72–0.88; *p *< 0.001), which remained consistent 0.84 (95% CI 0.75–0.93; *p *= 0.001) following further adjustment for several established and emerging risk factors. Following additional adjustment for hsCRP, the OR was 0.87 (95% CI 0.78–0.97; *p *= 0.012). In analyses that compared the top versus bottom tertiles of total bilirubin, the inverse associations remained except for evidence of attenuation to the null on further adjustment for hsCRP (Table 2).

**Table 3:** Association of baseline total bilirubin with incident NAFLD as measured by HSI

Total bilirubin level (µmol/l)	Events/total	Model 1		Model 2		Model 3		Model 4	
		OR (95% CI)	*p* value	OR (95% CI)	*p* value	OR (95% CI)	*p* value	OR (95% CI)	*p* value
Per 1 SD increase	452/3570	0.79 (0.72 to 0.88)	< 0.001	0.83 (0.75 to 0.93)	0.001	0.84 (0.75 to 0.93)	0.001	0.87 (0.78 to 0.97)	0.012
T1 (0.95–6)	229/1476	Ref.		Ref.		Ref.		Ref.	
T2 (7, 8)	107/967	0.67 (0.53 to 0.86)	0.002	0.75 (0.58 to 0.97)	0.026	0.77 (0.60 to 0.99)	0.041	0.80 (0.62 to 1.04)	0.094
T3 (≥ 9)	116/1127	0.63 (0.49 to 0.81)	< 0.001	0.71 (0.55 to 0.92)	0.009	0.73 (0.56 to 0.94)	0.014	0.78 (0.61 to 1.02)	0.066

2801 participants with prevalent NAFLD as measured by HSI were excluded

*CI* confidence interval, *HSI* hepatic steatosis index, *NAFLD* non-alcoholic fatty liver disease, *OR* odds ratio, *SD* standard deviation, *T* tertile

Model 1: Age and sex

Model 2: Model 1 plus smoking status, systolic blood pressure, total cholesterol, and high-density lipoprotein cholesterol

Model 3: Model 2 plus alcohol consumption, glucose, estimated glomerular filtration rate, and log_e_ urinary albumin excretion

Model 4: Model 3 plus log_e_ high-sensitivity C-reactive protein

The association between total bilirubin and NAFLD was not statistically significantly modified by sex for both measures of NAFLD (*p *> 0.05); whereas the association between total bilirubin and NAFLD (as estimated by FLI) was comparable in males and females, the age-adjusted inverse association between total bilirubin and NAFLD (as estimated by HSI) in males was attenuated to null on further adjustment for established risk factors (Table 4). The ORs for all associations remained similar in sensitivity analyses that involved exclusion of participants with prevalent diabetes, participants on cholesterol lowering medication, or participants with potential Gilbert’s syndrome (“Appendices 4–5”).

**Table 4:** Association of baseline total bilirubin with incident NAFLD in males and females

Gender	Events/total	Model 1		Model 2		Model 3		Model 4	
		OR (95% CI)	*p* value	OR (95% CI)	*p* value	OR (95% CI)	*p* value	OR (95% CI)	*p* value
*NAFLD as measured by Fatty Liver Index*									
Males	253/1551	0.71 (0.62 to 0.82)	< 0.001	0.80 (0.68 to 0.92)	0.002	0.79 (0.68 to 0.92)	0.002	0.81 (0.69 to 0.94)	0.007
Females	181/2273	0.71 (0.59 to 0.85)	< 0.001	0.75 (0.63 to 0.90)	0.002	0.75 (0.63 to 0.90)	0.002	0.83 (0.69 to 1.00)	0.055
*NAFLD as measured by Hepatic Steatosis Index*									
Males	358/1591	0.84 (0.72 to 0.97)	0.020	0.92 (0.80 to 1.08)	0.308	0.94 (0.81 to 1.09)	0.407	0.95 (0.81 to 1.11)	0.527
Females	91/1908	0.74 (0.64 to 0.86)	< 0.001	0.76 (0.65 to 0.88)	< 0.001	0.76 (0.65 to 0.88)	< 0.001	0.81 (0.69 to 0.94)	0.006

*CI* confidence interval, *NAFLD* non-alcoholic fatty liver disease, *OR* odds ratio, *SD* standard deviation, ORs are reported per 1-SD increase in log_e_ total bilirubin

Model 1: Age

Model 2: Model 1 plus smoking status, systolic blood pressure, total cholesterol, and high-density lipoprotein cholesterol

Model 3: Model 2 plus alcohol consumption, glucose, estimated glomerular filtration rate, and log_e_ urinary albumin excretion

Model 4: Model 3 plus log_e_ high-sensitivity C-reactive protein

### Mendelian randomization findings

There was strong evidence for an association between rs6742078 SNP and total bilirubin (0.70 SD increase in total bilirubin levels per T allele; SE = 0.03, *p *= 1.35 × 10^−80^) and the SNP explained 20% of total variance in total bilirubin levels. There was no evidence for associations between rs6742078 and confounders included in the observational analyses, except for a weak association with sex (“Appendix 6”). The causal OR for FLI was 0.98 (95% CI 0.69–1.38; *p *= 0.900) per 1 SD genetically elevated total bilirubin level and that for HSI was 1.14 (95% CI 0.81–1.59; *p *= 0.451) (Table 5).

**Table 5:** Causal estimates for NAFLD (as measured by FLI and HSI) using Mendelian randomisation analysis

NAFLD outcome	Events/total	Unadjusted OR (95% CIs)	*p* value	Adjusted^a^ OR (95% CIs)	*p* value
FLI	187/1610	0.98 (0.69 to 1.38)	0.900	0.99 (0.70 to 1.42)	0.978
HSI	190/1528	1.14 (0.81 to 1.59)	0.451	1.10 (0.79 to 1.54)	0.556

*CI* confidence interval, *FLI* fatty liver index, *HSI* hepatic steatosis index, *NAFLD* non-alcoholic fatty liver disease, *OR* odds ratio

^a^Model adjusted for age, sex, smoking status, systolic blood pressure, total cholesterol, high-density lipoprotein cholesterol, alcohol consumption, glucose, estimated glomerular filtration rate, log_e_ urinary albumin excretion, log_e_ high-sensitivity C-reactive protein

## Comments

### Key findings

Using a large-scale population-based study of predominantly Caucasian men and women without pre-existing disease including NAFLD at baseline, we have demonstrated that total bilirubin is inversely associated with future risk of NAFLD as estimated by two well-known biomarker indices, the FLI and HSI respectively. The associations were independent of several established risk factors and other potential confounders and remained robust in several sensitivity analyses. The inverse association between total bilirubin and NAFLD was not significantly modified by sex. However, given the rather relatively small numbers (low event rates) in males and females, larger-scale studies in both genders are needed to further evaluate a potential role of sex on the impact of bilirubin on new onset NAFLD. Furthermore, using a MR analysis, we investigated whether the observed association between total bilirubin and NAFLD was devoid of confounding and/or reverse causation. Despite strong observational evidence for associations between bilirubin and NAFLD, these results were not supported by MR analyses—there was no evidence for a causal relationship between genetically elevated bilirubin and NAFLD. While the rs6742078 SNP was strongly associated with levels of circulating bilirubin and the F statistic (> 400) indicated good instrument strength, the observed wide 95% CIs for the MR results are indicative of low power. For MR to be valid, several assumptions need to be met: [39] the genetic instrument (1) must be associated with the exposure of interest, (2) must not be associated with any confounders and (3) must not directly influence the outcome, except through the exposure of interest. When exploring the association between rs6742078 and confounding variables included in the observational analyses, no evidence for associations was found, except for weak evidence of an association between rs6742078 and sex. While this could be a chance finding, given the multiple tests that were performed, it is also possible that this might represent a true causal relationship. This might reflect the sex differences in hepatic uridine diphosphate glucuronyltransferase (UGT1A1) activity [40], the enzyme that contributes substantially to bilirubin glucuronidation and enhances bilirubin elimination. Consistent with our findings, previous studies have demonstrated higher levels of circulating bilirubin in men compared with women [19]. On the contrary, a number of studies have reported findings which suggest that the discordance in circulating bilirubin levels between males and females may not be attributable to differences in the UGT1A1 polymorphism [41–43]. Therefore, these findings deserve follow-up in larger cohort studies.

### Comparison with previous studies

A limited number of epidemiological observational studies have reported on the associations of bilirubin with the risk of NAFLD. In an analysis of over 17,000 participants, Kwak et al. demonstrated an inverse association between total bilirubin and NAFLD diagnosed on the basis of ultrasonographic findings and an alcohol consumption of less than 20 g/day [19]. However, the main limitation of this study was its cross-sectional design, which precluded the ability to assess the temporal relationship between bilirubin and the risk of NAFLD. Findings from two prospective cohort studies have demonstrated serum direct bilirubin to be associated with reduced risk of NAFLD, but found no evidence of associations for total or indirect bilirubin [17, 20]. To our knowledge, this is the first study to demonstrate a long-term prospective association between total bilirubin and the risk of NAFLD in a general predominantly Caucasian population. We are also the first to investigate whether a potential causal association exists between elevated total bilirubin levels and decreased NAFLD risk in a general adult population. Furthermore, our study employed NAFLD outcomes diagnosed on the basis of biomarker-based indices, whereas previous relevant studies have utilized ultrasonographic findings [17–20].

### Potential explanations for findings

Bilirubin, iron and carbon monoxide are degradation products of heme catabolism via heme oxygenase-1 (HO-1) and they have been reported to regulate important functions in cells [44]. Increasing evidence suggests that bilirubin has cytoprotective properties [45], antioxidant actions [13, 14] and anti-inflammatory effects via its anti-complement actions [15, 46]. Since oxidative stress mechanisms and inflammation play a major role in the pathophysiology of NAFLD [47, 48], if there is a protective effect of bilirubin on NAFLD risk, this may be via the potent antioxidant and anti-inflammatory effects of bilirubin. Whether total bilirubin is a direct cause of NAFLD or just a risk marker of underlying NAFLD, is not certain at the moment. Our MR findings did not provide evidence for a causal association between circulating total bilirubin and NAFLD. It may be possible that the observational association may be driven by biases such as unmeasured confounding and/or reverse causation. The current evidence suggests that total bilirubin may just be a risk marker of NAFLD. However, given that the MR estimates were under-powered and a causal effect of bilirubin on NAFLD cannot be completely ruled out, further investigation in a larger sample is necessary.

### Implications of findings

The current findings further contribute to the accumulating body of evidence on the putative protective role of bilirubin on adverse cardiometabolic outcomes such as hypertension, diabetes, metabolic syndrome and CVD [1–4]. Indeed, the utility of using circulating bilirubin to prevent and treat cardiometabolic disease has been extensively debated [23–25]. It is reported that patients with Gilbert syndrome (characterised by moderate hyperbilirubinaemia) have reduced levels of markers of oxidative stress and inflammation and have decreased risk of vascular complications; [49, 50] hence, it is possible that the sustained hyperbilirubinaemia prevents vascular complications via inhibition of oxidative stress and inflammation. Circulating bilirubin is a simple, standardised, cheap, and easily measured biomarker, hence its potential value in reducing the risk of adverse cardiometabolic outcomes warrants further evaluation.

### Strengths and limitations

In addition to the novelty, several strengths of this work deserve mention. These include employing a sample that was representative of the general population, exclusion of participants with pre-existing disease which minimised biases due to reverse causation, accounting for several key clinical characteristics, and conducting several sensitivity analyses to confirm the robustness of the findings. In our MR analysis, we used the rs6742078 variant to examine the potential causal relationship, which has been shown to be strongly associated with substantial increases in levels of bilirubin and explained substantial proportion of variation in bilirubin levels. The limitations of the current work include the inability to generalise the findings to other populations as the sample predominantly comprised of white adults of Dutch descent, absence of data on repeat measurements of bilirubin to correct for regression dilution bias, and inability to replicate or verify the findings in an independent cohort due to lack of data. One might say that the association between rs6742078 and sex violated one of the conditions for the MR analysis, hence impacting on the causal estimates. However, this is unlikely for the following reasons: (1) the association was weak and (2) it is very likely this was a chance finding (due to the multiple statistical tests) given that the rs6742078 variant has consistently been demonstrated to be very specific for bilirubin and is not associated with various demographics (including sex), lifestyle and clinical variables in several MR studies [5, 6, 30] and including the PREVEND cohort [4] which was employed for these analyses. In addition, the power to detect an effect similar to the observational estimates was low and larger samples (with higher number of cases) are required. In addition, the availability of large samples for genome-wide association study, such as UK Biobank [51], will increase the discovery of genetic variants, which will in turn provide better instruments for MR analyses.

## Conclusion

Elevated circulating total bilirubin was independently associated with decreased risk of new onset NAFLD in a cohort of apparently healthy predominantly Caucasian men and women without pre-existing CVD or NAFLD. Based on MR analysis, there was little evidence to suggest a causal association. The observational association may be driven by biases such as unmeasured confounding and/or reverse causation. However, due to low statistical power, larger-scale investigations are necessary to draw definitive conclusions.

## Appendix 1

See Table 6.

**Table 6:** STROBE 2007 Statement—Checklist of items that should be included in reports of cohort studies

Section/topic	Item #	Recommendation	Reported on page #
*Title and abstract*	1	(a) Indicate the study’s design with a commonly used term in the title or the abstract	Page 1
		(b) Provide in the abstract an informative and balanced summary of what was done and what was found	Page 2
*Introduction*			
Background/rationale	2	Explain the scientific background and rationale for the investigation being reported	Page 3
Objectives	3	State specific objectives, including any prespecified hypotheses	Page 3–4
*Methods*			
Study design	4	Present key elements of study design early in the paper	Study population
Setting	5	Describe the setting, locations, and relevant dates, including periods of recruitment, exposure, follow-up, and data collection	Study population
Participants	6	(*a*) Give the eligibility criteria, and the sources and methods of selection of participants. Describe methods of follow-up	Study population
		(*b*) For matched studies, give matching criteria and number of exposed and unexposed	Not applicable
Variables	7	Clearly define all outcomes, exposures, predictors, potential confounders, and effect modifiers. Give diagnostic criteria, if applicable	Risk Factor Assessment
Data sources/measurement	8*	For each variable of interest, give sources of data and details of methods of assessment (measurement). Describe comparability of assessment methods if there is more than one group	Risk Factor Assessment
Bias	9	Describe any efforts to address potential sources of bias	Statistical Methods
Study size	10	Explain how the study size was arrived at	Statistical Methods
Quantitative variables	11	Explain how quantitative variables were handled in the analyses. If applicable, describe which groupings were chosen and why	Statistical Methods
Statistical methods	12	(a) Describe all statistical methods, including those used to control for confounding	Statistical Methods
		(b) Describe any methods used to examine subgroups and interactions	Statistical Analyses
		(c) Explain how missing data were addressed	Not applicable
		(d) If applicable, explain how loss to follow-up was addressed	Not applicable
		(e) Describe any sensitivity analyses	Statistical Methods
*Results*			
Participants	13*	(a) Report numbers of individuals at each stage of study—e.g. numbers potentially eligible, examined for eligibility, confirmed eligible, included in the study, completing follow-up, and analysed	Study population
		(b) Give reasons for non-participation at each stage	Study population
		(c) Consider use of a flow diagram	
Descriptive data	14*	(a) Give characteristics of study participants (e.g. demographic, clinical, social) and information on exposures and potential confounders	Results; Table 1; “Appendices 2–3”
		(b) Indicate number of participants with missing data for each variable of interest	
		(c) Summarise follow-up time (e.g. average and total amount)	Results
Outcome data	15*	Report numbers of outcome events or summary measures over time	Results
Main results	16	(*a*) Give unadjusted estimates and, if applicable, confounder-adjusted estimates and their precision (e.g. 95% confidence interval). Make clear which confounders were adjusted for and why they were included	Results; Tables 2, 3 and 4
		(*b*) Report category boundaries when continuous variables were categorized	Results; Tables 2, 3 and 4
		(*c*) If relevant, consider translating estimates of relative risk into absolute risk for a meaningful time period	
Other analyses	17	Report other analyses done—e.g. analyses of subgroups and interactions, and sensitivity analyses	Results; “Appendices 4–5”
*Discussion*			
Key results	18	Summarise key results with reference to study objectives	Discussion—Summary of main findings
*Limitations*			
Interpretation	20	Give a cautious overall interpretation of results considering objectives, limitations, multiplicity of analyses, results from similar studies, and other relevant evidence	Discussion
Generalisability	21	Discuss the generalisability (external validity) of the study results	Discussion
*Other information*			
*Funding*	22	Give the source of funding and the role of the funders for the present study and, if applicable, for the original study on which the present article is based	Pages 14–15

## Appendix 2

See Table 7.

**Table 7:** Baseline participant characteristics according to the development of NAFLD as measured by FLI

	Without NAFLD (N = 3390) Mean (SD) or median (IQR) or n (%)	With NAFLD (N = 434) Mean (SD) or median (IQR) or n (%)	*p* value*
Total bilirubin (µmol/l)	7 (5–9)	7 (5–9)	< 0.001
*Questionnaire*			
Males	1298 (38.3)	253 (58.3)	< 0.001
Age at survey (years)	47 (12)	50 (12)	< 0.001
History of diabetes	18 (0.5)	5 (1.2)	0.115
Current and former smokers	2212 (65.3)	303 (69.8)	0.059
Alcohol consumers	2606 (76.9)	332 (76.5)	0.861
*Physical measurements*			
BMI (kg/m^2^)	24.1 (2.7)	27.1 (2.5)	< 0.001
Waist circumference (cm)	81.5 (9.3)	90.9 (7.0)	< 0.001
SBP (mmHg)	122 (17)	131 (18)	< 0.001
DBP (mmHg)	71 (9)	75 (9)	< 0.001
*Lipid, metabolic, inflammatory, liver, and renal markers*			
Total cholesterol (mmol/l)	5.40 (1.07)	5.80 (1.02)	< 0.001
HDL-C (mmol/l)	1.45 (0.39)	1.22 (0.32)	< 0.001
Triglycerides (mmol/l)	0.95 (0.73–1.26)	1.23 (0.96–1.61)	< 0.001
Glucose (mmol/l)	4.57 (0.72)	4.85 (0.76)	< 0.001
GGT (U/L)	18 (14–25)	26 (19–36)	< 0.001
ALT (U/L)	18 (14–23)	21 (16–28)	< 0.001
AST (U/L)	23 (20–26)	24 (21–28)	< 0.001
hsCRP (mg/l)	0.84 (0.38–1.96)	1.49 (0.75–3.05)	< 0.001
eGFR (ml/min/1.73 m^2^)	91.2 (15.0)	87.3 (15.0)	< 0.001
UAE (mg/24 h)	7.99 (5.84–12.30)	9.42 (6.53–14.61)	< 0.001

*ALT* alanine aminotransferase, *AST* aspartate aminotransferase, *BMI* body mass index, *DBP* diastolic blood pressure, *eGFR* estimated glomerular filtration rate (as calculated using the Chronic Kidney Disease Epidemiology Collaboration combined creatinine-cystatin C equation), *FLI* fatty liver index, *GGT* gamma-glutamyltransferase, *HDL-C* high-density lipoprotein cholesterol, *hsCRP* high-sensitivity C-reactive protein, *NAFLD* non-alcoholic fatty liver disease

*Employed a two-sample *t*-tests for a difference in means for continuous variables and a Chi square test for categorical variables

## Appendix 3

See Table 8.

**Table 8:** Baseline participant characteristics according to the development of NAFLD as measured by HSI

	Without NAFLD (N = 3118) Mean (SD) or median (IQR) or n (%)	With NAFLD (N = 452) Mean (SD) or median (IQR) or n (%)	*p* value*
Total bilirubin (µmol/l)	7 (6–9)	6 (5–9)	< 0.001
*Questionnaire*			
Males	1432 (45.9)	195 (43.1)	0.266
Age at survey (years)	47 (12)	48 (12)	0.101
History of diabetes	11 (0.4)	2 (0.4)	0.767
Current and former smokers	2116 (67.9)	306 (67.7)	0.944
Alcohol consumers	2459 (78.9)	322 (71.2)	< 0.001
*Physical measurements*			
BMI (kg/m^2^)	23.7 (2.4)	26.1 (2.0)	< 0.001
Waist circumference (cm)	81.8 (10.0)	87.9 (9.5)	< 0.001
SBP (mmHg)	123 (18)	128 (19)	< 0.001
DBP (mmHg)	72 (9)	73 (9)	< 0.001
*Lipid, metabolic, inflammatory, liver, and renal markers*			
Total cholesterol (mmol/l)	5.43 (1.08)	5.69 (1.10)	< 0.001
HDL-C (mmol/l)	1.43 (0.40)	1.33 (0.38)	< 0.001
Triglycerides (mmol/l)	0.99 (0.74–1.36)	1.15 (0.85–1.63)	< 0.001
Glucose (mmol/l)	4.58 (0.70)	4.74 (1.07)	< 0.001
GGT (U/L)	19 (14–27)	21 (14–31)	< 0.001
ALT (U/L)	17 (14–22)	19 (15–25)	< 0.001
AST (U/L)	23 (20–27)	23 (20–28)	0.023
hsCRP (mg/l)	0.84 (0.37–1.99)	1.26 (0.62–2.69)	< 0.001
eGFR (ml/min/1.73 m^2^)	90.8 (15.3)	88.9 (15.1)	0.016
UAE (mg/24 h)	8.28 (5.96–13.13)	8.57 (6.13–12.91)	0.423

*ALT* alanine aminotransferase, *AST* aspartate aminotransferase, *BMI* body mass index, *DBP* diastolic blood pressure, *eGFR* estimated glomerular filtration rate (as calculated using the Chronic Kidney Disease Epidemiology Collaboration combined creatinine-cystatin C equation), *GGT* gamma-glutamyltransferase, *HDL-C* high-density lipoprotein cholesterol, *hsCRP* high-sensitivity C-reactive protein, *HSI* hepatic steatosis index, *NAFLD* non-alcoholic fatty liver disease

*Employed a two-sample *t*-tests for a difference in means for continuous variables and a Chi square test for categorical variables

## Appendix 4

See Table 9.

**Table 9:** Association of baseline serum total bilirubin levels with FLI in several sensitivity analyses

	Events/total	Model 1		Model 2		Model 3		Model 4	
		OR (95% CI)	*p* value	OR (95% CI)	*p* value	OR (95% CI)	*p* value	OR (95% CI)	*p* value
Exclusion of people with diabetes at baseline	429/3801	0.71 (0.63 to 0.79)	< 0.001	0.77 (0.69 to 0.86)	< 0.001	0.77 (0.68 to 0.86)	< 0.001	0.81 (0.72 to 0.92)	0.001
Exclusion of people on cholesterol lowering medication	416/3713	0.71 (0.64 to 0.80)	< 0.001	0.77 (0.69 to 0.87)	< 0.001	0.77 (0.68 to 0.87)	< 0.001	0.82 (0.73 to 0.92)	0.001
Exclusion of people with potential Gilbert’s disease	434/3820	0.72 (0.64 to 0.80)	< 0.001	0.78 (0.69 to 0.88)	< 0.001	0.78 (0.69 to 0.87)	< 0.001	0.82 (0.73 to 0.93)	0.001

Gilbert’s disease was defined as total bilirubin > 34.2 µmol/L, aspartate aminotransferase < 80 IU/L, alanine aminotransferase < 80 IU/L, and gamma-glutamyltransferase < 80 IU/L

2420 participants with prevalent NAFLD as measured by FLI were excluded

*CI* confidence interval, *FLI* fatty liver index, *NAFLD* non-alcoholic fatty liver disease, *OR* odds ratio, *SD* standard deviation, *T* tertile

Model 1: Age and sex

Model 2: Model 1 plus smoking status, systolic blood pressure, total cholesterol, and high-density lipoprotein cholesterol

Model 3: Model 2 plus alcohol consumption, glucose, estimated glomerular filtration rate, and log_e_ urinary albumin excretion

Model 4: Model 3 plus log_e_ high-sensitivity C-reactive protein

## Appendix 5

See Table 10.

**Table 10:** Association of baseline serum total bilirubin levels with HSI in several sensitivity analyses

	Events/total	Model 1		Model 2		Model 3		Model 4	
		OR (95% CI)	*p* value	OR (95% CI)	*p* value	OR (95% CI)	*p* value	OR (95% CI)	*p* value
Exclusion of people with diabetes at baseline	450/3557	0.79 (0.71 to 0.88)	< 0.001	0.83 (0.75 to 0.92)	0.001	0.84 (0.75 to 0.93)	0.001	0.87 (0.78 to 0.97)	0.011
Exclusion of people on cholesterol lowering medication	430/3453	0.81 (0.73 to 0.90)	< 0.001	0.86 (0.77 to 0.96)	0.005	0.87 (0.77 to 0.96)	0.008	0.89 (0.80 to 1.00)	0.041
Exclusion of people with potential Gilbert’s disease	452/3566	0.79 (0.72 to 0.88)	< 0.001	0.83 (0.75 to 0.93)	0.001	0.84 (0.75 to 0.94)	0.002	0.87 (0.78 to 0.97)	0.014

Gilbert’s disease was defined as total bilirubin > 34.2 µmol/L, aspartate aminotransferase < 80 IU/L, alanine aminotransferase < 80 IU/L, and gamma-glutamyltransferase < 80 IU/L

2801 participants with prevalent NAFLD as measured by HSI were excluded

*CI* confidence interval, *HSI* hepatic steatosis index, *NAFLD* non-alcoholic fatty liver disease, *OR* odds ratio, *SD* standard deviation, *T* tertile

Model 1: Age and sex

Model 2: Model 1 plus smoking status, systolic blood pressure, total cholesterol, and high-density lipoprotein cholesterol

Model 3: Model 2 plus alcohol consumption, glucose, estimated glomerular filtration rate, and log_e_ urinary albumin excretion

Model 4: Model 3 plus log_e_ high-sensitivity C-reactive protein

## Appendix 6

See Table 11.

**Table 11:** Associations between rs6742078 and confounders

Confounders	β coefficients (95% CIs)	*p* value
Age	− 0.05 (− 0.94, 0.85)	0.919
Male sex	− 0.04 (− 0.08, 0.00)	0.027
Smoking	0.00 (− 0.04, 0.04)	0.966
Systolic blood pressure	1.13 (− 0.19, 2.46)	0.093
Total cholesterol	− 0.01 (− 0.09, 0.07)	0.855
HDL-cholesterol	− 0.01 (− 0.04, 0.02)	0.353
Alcohol consumption	− 0.01 (− 0.04, 0.02)	0.483
Fasting glucose	0.00 (− 0.05, 0.06)	0.932
Estimated GFR	0.27 (− 0.87, 1.42)	0.638
UAE	− 0.02 (− 0.08, 0.04)	0.561
hsCRP	− 0.07 (− 0.16, 0.02)	0.109

Analysis was based on 1610 participants

*GFR* glomerular filtration rate, *hsCRP* high-sensitivity C-eactive protein, *UAE* urinary albumin excretion, *HDL* high-density lipoprotein
